# A study protocol for the policy intervention design and development of the implementation strategies for direct access to physiotherapists in primary care: a sequential mixed-method study using implementation mapping and a Delphi survey

**DOI:** 10.1186/s43058-024-00680-y

**Published:** 2024-12-18

**Authors:** Eng Kiong Yeoh, Carrie Ho Kwan Yam, Ethan Ming Yin Ip, Tsz Yu Chow, Chi Tim Hung

**Affiliations:** The Centre for Health Systems and Policy Research, Jockey Club School of Public Health and Primary Care, The Chinese University of Hong Kong, Hong Kong, China

**Keywords:** Direct physiotherapist access, Implementation mapping, Implementation research logic model, Intervention characteristics, Policy design, Determinants, Implementation strategies

## Abstract

**Background:**

In many Asian jurisdictions, patients are required to obtain referrals from registered doctors before consulting physiotherapists. In contrast, countries such as the United States, the United Kingdom, and Australia have a direct access model for physiotherapists designed across different healthcare settings and under prescribed conditions. While research has demonstrated the benefits of direct access, issues remain on the appropriate policy design for direct access in the context of patient safety and organizational challenges in the implementation. Recently the policy to allow direct access in primary care context is being considered in Hong Kong. This study aims to examine the intervention design options for the policy of direct access to physiotherapists and identify corresponding implementation strategies, to inform the appropriate intervention design for direct access to physiotherapists and the implementation strategies.

**Methods:**

We adopt a systematic process for developing the design of the policy and the implementation strategies using an Implementation Mapping approach informed by Consolidated Framework for Implementation Research (CFIR). We will conduct literature reviews to understand the different aspects of policy intervention design and employ qualitative in-depth interviews and focus group discussions to understand key stakeholders’ perspectives related to the direct access model. The identified barriers and facilitators associated with policy implementation of an acceptable intervention design will inform the development of an effective implementation strategy tailored to the implementation context. Our approach will involve mapping the research evidence and the subsequent findings from the stakeholders’ deliberations into the CFIR domains and referencing the Expert Recommendations for Implementing Change (ERIC) to develop the acceptable intervention characteristics and the corresponding implementation strategies. These insights will be further validated in a Delphi Expert Survey, for a consensus-based approach.

**Discussion:**

This study employs a sequential mixed-method approach to explore the intervention characteristics for an acceptable intervention design in the policy formulation and the corresponding implementation strategy for direct access to physiotherapists. Integrating research insights into actionable policy recommendations and refining these recommendations in a Delphi Survey will inform the appropriate policy intervention design and implementation strategy for direct access to physiotherapy services.

**Supplementary Information:**

The online version contains supplementary material available at 10.1186/s43058-024-00680-y.

Contributions to the literature’ section • Challenges exist in achieving consensus on acceptable policy intervention design and actionable implementation strategies for direct access to physiotherapists due to patient safety concerns.

• We will employ an Implementation Mapping approach, leveraging the Consolidated Framework for Implementation Research and Expert Recommendations for Implementation Change taxonomy, to outline the steps for developing policy design options and implementation strategies.

• Our study will contribute to the existing knowledge-base by demonstrating the effective integration of implementation science and a deliberative process involving expert stakeholders through the Delphi method for designing acceptable evidenced-informed policy interventions and implementation strategies.

## Background

In the primary healthcare setting, providing access and strengthening the role and competence of different healthcare professionals in the healthcare system is crucial for improving healthcare quality and meeting patients’ needs. Primary healthcare is the first point of contact for individuals and families in a healthcare delivery system in their living and working communities, encompassing the provision of accessible, comprehensive, continuous, coordinated, and person-centered care [1]. Traditionally, primary care physicians serve as the first contact point in most healthcare systems, responsible for referring patients to secondary care and other healthcare professionals such as physiotherapists if indicated. “Direct access to healthcare professional services”, also known as “self-referral” [2], allows patients to seek therapy without requiring an initial consultation with another healthcare professional. Demographic shifts, such as an ageing population and the associated rising prevalence of chronic diseases, including musculoskeletal disorders, have prompted a shift toward enabling self-referral to physiotherapists to improve access to timely treatment and management of musculoskeletal disorders. In the United Kingdom (UK), allied health professionals have been able to act as first contact practitioners since 1978 and is well established in the private sector. However, the practice is not universal in the national health sectors with inconsistency in access across the country. In 2008, the UK’s Department of Health piloted a model for self-referral to physiotherapy to evaluate the effect on access, demand, patient outcomes and health professionals experiences [2]. Similarly, many countries have implemented programs enabling patients to directly access physiotherapists [3]. A systematic review and meta-analysis in the US finding that direct access to physiotherapists is cost-effective, resulting in fewer visits compared to physician-first access, along with greater functional improvement [4]. However there were a number of limitations in the systematic review, only 5 studies were eligible spanning 1997 to 2019 and only 2 studies for between groups analysis for functional outcomes and costs. Another systematic review highlighted the efficiency gains associated with direct access physiotherapy [5]. However the general quality of the evidence was low and the authors advised caution of the findings The World Physiotherapy Organization [3] also advocates for direct access to physiotherapists, emphasizing improved clinical outcomes. Despite these benefits, concerns persist regarding patient safety. Physicians continue to raise questions about physiotherapists’ competencies in differential diagnosis, and the ability to recognize medical ‘red flags’, signs symptoms of diseases that require referral to other healthcare professionals, as well as the fear of de-skilling physicians in musculoskeletal diagnosis and management [6, 7].

However, the intervention design of the policy allowing direct access to physiotherapists varies significantly across different jurisdictions, differing in the practice model, nature of diseases permitted, provider settings and financing model. In some countries, including the UK, Australia, Singapore, and Thailand, direct access to physiotherapists is permitted as long as the conditions fall within the scope of practice [3]. In the US, provisions for direct access differ across states, from unlimited access to access with provisions and limits on time and visits [8]. In contrast, other health systems such as in Denmark, France, Italy, the Netherlands, Macau Special Administrative Region, and Indonesia direct access is restricted to the private sector only [9]. Notably, certain jurisdictions, such as the Hong Kong Special Administrative Region, Germany, Japan, and Korea do not allow physiotherapists to take on patients without prior physician referral.

Bury and Strokes identified key facilitators for the successful implementation of direct access to physiotherapists [10]. These include enhancing patients’ knowledge and education, equipping physiotherapists with necessary competencies to accept patients who directly seek their services, recognizing physiotherapy as an autonomous profession through legislation, and strong professional organizational leadership committed to achieving the goal of direct patient access [10, 11]. In contrast, a systematic review by Babatunde et al. highlighted perceived barriers related to patient safety [6]. These barriers include concerns about physiotherapists’ competence in medical screening and differential diagnosis, which could impact overall medical resource utilization. Additionally, contextual organizational challenges exist, such as insufficient knowledge among healthcare providers and administrators regarding direct access in ambulatory settings, legal considerations, risk management policies, and facility-specific requirements [6]. When designing the intervention for direct access, policymakers need to consider various factors carefully, including legislative frameworks, preconditions, the desired professional practice model and the acceptability of the policy intervention design. Furthermore, health system contextual factors health service funding models and reimbursement policies significantly influence the feasibility and effectiveness of direct access [10].

In Hong Kong, the Codes of Conduct outlined in the Supplementary Medical Professionals Ordinance currently mandate that physiotherapists and occupational therapists can only assess and/or treat patients upon referral from a registered doctor [12, 13]. Given the benefits of direct access care for patients, the physiotherapy profession in Hong Kong has advocated for the direct access model [14]. However, medical doctors hold divergent views on the direct access model [15, 16]. The complexity of this issue stems from concerns about physiotherapists’ competencies, and knowledge gaps and vested professional interests, which pose significant challenges in arriving at a consensus for the design of the intervention and implementation of a direct access model. Recognizing the need to improve patient access and reduce delays in appropriate healthcare, the Hong Kong Government has announced the intention to propose legislative amendments which will enable patients direct access to allied health professionals, including physiotherapists and occupational therapists under prescribed conditions, without requiring a doctor’s referral [17, 18]. However even when the legal constraints through changes in the ordinance have been removed, the long-established practice of patients seeking care primarily from physicians first may still deter them from seeking care directly from physiotherapists. Therefore, studying the options in the practice model for the intervention design for the practice model of direct access and the corresponding implementation strategies is crucial for its adoption and effectiveness.

This study aims to examine the design of the policy intervention, including the conditions and settings for permitting direct access, by referencing international literature and countries’ experiences. We will also assess key stakeholders’ perspectives and views on the acceptability of the options for the appropriate practice model for direct access to physiotherapists, and exploring corresponding barriers and facilitators associated with policy implementation. We will use an Implementation Mapping (IM) approach [19], Consolidated Framework for Implementation Research (CFIR) [20], and Expert Recommendations for Implementing Change (ERIC) methodologies [21], and verified through a Delphi Survey to inform the development of an acceptable policy intervention design and effective implementation strategy and actions needed to achieve the policy objective in Hong Kong.

## Methods

### Study conceptual framework

**Fig. 1:**
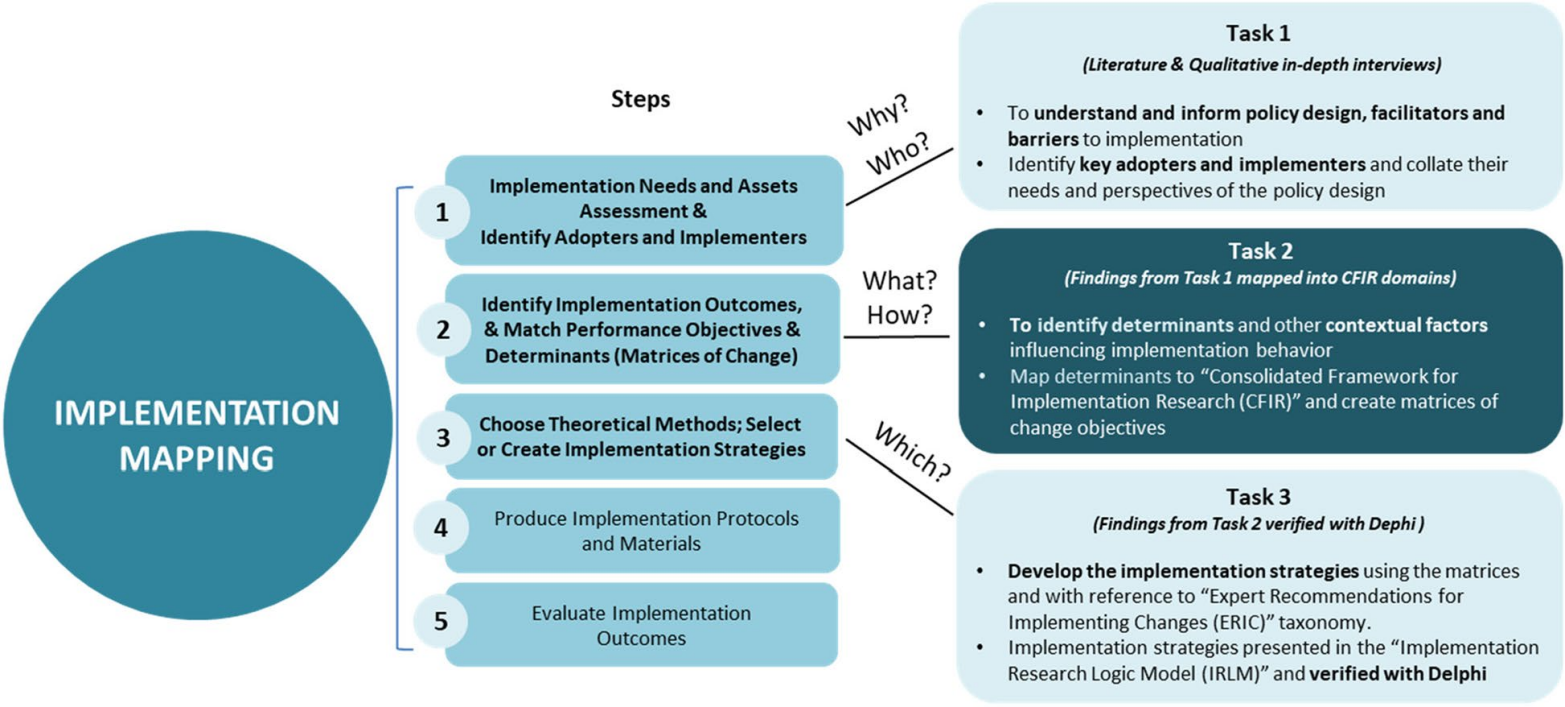
Study conceptual framework

The effectiveness and impact of direct access to physiotherapists depends not only on the decision in the formulation of an appropriate and acceptable policy design of the intervention, but also on its successful implementation, completeness, fidelity, and reach within the targeted population. Implementation Science facilitates the translation of research evidence on the benefit of direct access demonstrated in the literatures into effective practice and policy [19]. To successfully implement the policy for direct access in Hong Kong, we will first develop the intervention design options for the policy of direct access in a deliberative process involving the key stakeholders informed by the research evidence in a multi-staged mixed method study. Secondly, we will systematically develop the implementation strategies by applying the IM approach, CFIR, and ERIC as the study’s conceptual framework (Fig. 1 and Additional File 1).

### Study design

The multi-staged mixed method design comprise:

(i) a literature review to synthesize the elements and characteristics of the policy intervention for direct access to physiotherapists and the contexts of their implementation, the barriers and facilitators encountered and the effects and outcomes of the practice; and
(ii) an iterative deliberative process of experts and key stakeholders to (a) appraise and interpret the research findings and the application of the practice models in the policy intervention and (b) assess the appropriateness and acceptability of the policy design and the barriers and facilitators in implementation.

#### *Formation of expert steering group*

Given the specialized nature of this topic, we will establish an Expert Steering Group which shall comprise four experts including two experienced physiotherapists (one each from the public and private sector), and two experienced doctors (specializing in Family Medicine and Orthopedics). This Expert Steering Group will play a pivotal role in developing the options for the policy intervention design and the subsequent implementation strategies for physiotherapy direct access. Their responsibilities include assessing the research evidence and gaps, the applicability and acceptability of different practice models for direct access in the local context, and issues and barriers in implementation. Regular meetings will be conducted to collaboratively develop an Implementation Research Logic Model (IRLM) which define outcomes, objectives, and determinants, and discuss findings.

#### *Task 1: A needs assessment to identify all actors by their roles, and potential barriers and facilitators in implementation*

This task will involve two primary components: (i) literature and documentary review to understand policy design, including legislation and policy mandates, the scope and limits of practice, conditions for direct access, and implementation strategies, and (ii) qualitative in-depth interviews and focus groups with key stakeholders to identify key adopters and implementers and collate their perspectives on the needs for direct access and the policy design.

### Literature review

A structured literature search will be performed to systematically collect information on policy and design. Specifically, we will examine legislation and policy mandates related to direct access, the scope and limits of practice, and conditions imposed and factors affecting implementation. Our search will extend to international experiences, focusing on countries where direct access has been implemented, and examine guidelines and measures to safeguard patient safety in such contexts. Information sources include international health agencies, such as the World Health Organization (WHO) and World Physiotherapy Association, academic journal articles, government documents, and other relevant grey literature will also be included and analyzed. Case studies from countries with health systems governance for health professions similar to Hong Kong (e.g., the UK, Australia, and Singapore) will provide valuable insights for the appropriate policy design such as any conditions imposed and criteria for direct access. The literature review will play a crucial role in translating policy insights into policy design and actionable elements.

### Qualitative in-depth interviews and focus groups

Qualitative research is a distinct method to collect data including key informant interviews and focus group discussions. The use of qualitative methods intends to gain an in-depth understanding of the perspectives of adopters, implementers, and other stakeholders on the direct access model.

#### (a) Key informant interviews

Key informant interviews will be conducted within the first two months of the study to understand stakeholders’ perspectives on policy design for direct access. We will explore parameters and components and characteristics of the intervention, such as criteria/ conditions for direct access, considering the local context, legislative changes, and resource requirements. We will also examine the roles of different stakeholders in policy implementation, such as understanding “who has to do what?”, and identifying anticipated barriers and facilitators for implementation. These may include necessary skills or training required, patients’ knowledge, and concerns from other professions. Institutional commitment and leadership, crucial for organizational change during implementation, will also be explored. If needed, a second round of interviews will be conducted to seek input from policymakers and medical and physiotherapy professionals on the proposed implementation strategies developed.

In addition to policy decision makers, subjects for key informant interviews will involve all main stakeholders in the study, with the following inclusion criteria: (1) individuals who can effectively communicate their experience and opinions, or (2) those knowledgeable about policies for physiotherapy practice, or (3) professionals with relevant experience in physiotherapy or related fields, or (4) users of physiotherapy services. As with all qualitative research, the sample size will be ultimately be determined by data saturation [22]. We will use a maximum variation sampling strategy to enhance the heterogeneity and diversity of the interviewees [23], including key government officials, representatives from the Physiotherapists Board, the Academy of Medicine, physiotherapy associations, medical associations, provider organizations, insurance associations, tertiary education institutes offering physiotherapy programs, and representatives from patient groups. Approximately 20 key informants will be interviewed, with the number expanding until data saturation is reached.

Each interview will be conducted by the principal investigator and co-investigators, and last approximately 60 min. Tailored interview guides will be developed and pilot-tested for different categories of interviewees. Participants will provide informed consent and grant permission for audio recordings. Verbatim transcription of interview recordings will occur immediately after each session.

#### (b) Focus group discussions

Focus group discussions will also be conducted with the relevant stakeholders, particularly frontline professionals, to understand their knowledge, attitudes, and skills for direct access, and to identify barriers and facilitators for effective implementation. The approach is inductive, guided by thematic analysis [24]. Focus group discussions and semi-structured individual interviews will be conducted following the qualitative research approach [25]. The questions will include views on the determinants and necessary components for direct access, including the intervention characteristics, required training and support, professional and patient capabilities, and other contextual factors taking reference from the constructs in the CFIR framework.

Subjects for the focus group will include three categories of participants: (1) adopters (i.e., patients who have ever consulted physiotherapists), (2) providers (i.e., physiotherapists from both public and private sectors), and (3) referrers (i.e., medical doctors who refer patients to physiotherapy services). Quota sampling will be used to ensure a mix of certain important characteristics of the participants, including those from public and private sectors, hospital and community settings, and different specialties such as orthopedics, family medicine, neurology, cardiology, etc. [23]. To enable in-depth conversations to understand lived experiences of patients and healthcare practitioners’ perspectives, we will include only 4–6 participants for each focus group [26, 27]. In total, nine focus groups will be held, with an expected number of participants ranging from 36 to 54.

Tailored focus group discussion protocols will be developed for each group, with a set of open-ended questions (and the probe for responses) based on relevant literature and in-depth interviews [10, 11]. For patient groups, the focus will be on their experiences using physiotherapist services, including reasons for using the service, referral experiences, patient autonomy, choice of service provider, service quality, availability, accessibility, efficiency, accountability, and transparency. Additionally, we will explore their views on direct access, considering service demand and any safety and any other concerns they may have. For physiotherapist groups, we aim to understand their perceived roles in healthcare and perspectives on direct access to physiotherapy, including the criteria and parameters for direct access. Discussion topics will include service demand, the readiness of physiotherapists (including their knowledge base and ability to identify sinister events for referral back to doctors), and anticipated facilitators or barriers in implementing direct access (including entry-level education preparation, continuous training, and support required). For medical doctors, their referral practice, attitudes toward direct access, and considerations related to patient safety, accountability, oversight, and control of professional practice will be explored. Their views on the criteria and parameters for direct access and the potential impact of direct access will be sought.

To maximize variation and comprehensiveness of data derived from the discussions, focus groups will be held separately for each group, as each group may perceive their roles differently. Discussions will continue until no new relevant data emerges within each group. If scheduling conflicts arise, individual interviews will be arranged.

Each focus group discussion will be facilitated by the trained co-investigators to lead the discussion, and a note-taker to observe, facilitate, and capture key points of the discussion. Discussions will last approximately 90–120 min and will be audio-taped and transcribed verbatim. Notes taken during the discussion and from the debriefing session afterward will also be included in data analysis. Informed consent will be obtained from each participant before the discussion. All participants will complete a short questionnaire survey at the beginning of the session, covering basic demographic information and characteristics related to group division (e.g., type of patients, or working in the public or private sector).

##### *Task 2: To define implementation outcomes, performance objectives, determinants, and change objectives*

Initially, patients are considered adopters who make decisions regarding direct access practice, while physiotherapists and provider organizations act as implementers, delivering services based on agreed conditions and criteria. Based on findings from Task 1, we will define implementation outcomes, objectives, and determinants. Implementation outcomes include various aspects, including acceptability, adoption, appropriateness, costs, feasibility, fidelity, penetration, and sustainability of the policy [25]. Performance objectives specify the necessary implementation behaviors required to achieve an implementation outcome, addressing the fundamental question: “Who needs to do what?” to successfully adopt and implement the direct access model. We will use the qualitative findings from Task 1, mapped to the CFIR, to identify relevant personal determinants and other contextual factors influencing implementation behaviors for adopters and implementers. Additionally, we will seek input from the expert steering group in identifying the policy design options as the intervention characteristics and creating the matrices of change objectives, specifying the necessary changes in each determinant to achieve the corresponding implementation behavior. By systematically defining these elements, we aim to inform the design of implementation strategies in Task 3.

##### *Task 3: To develop the design of the policy intervention and implementation strategies, verified by a Delphi survey*

The matrices of change objectives derived from Task 2 will guide the development of implementation strategies. To categorize these strategies effectively, we will reference the ERIC taxonomy, which encompasses 73 discrete implementation strategies grouped into nine thematic clusters [21, 28]. These clusters include (1) use evaluative and iterative strategies, (2) provide interactive assistance, (3) adapt and tailor to context, (4) develop stakeholder interrelationships, (5) train and educate stakeholders, (6) support clinicians, (7) engage consumers, (8) utilize financial strategy, and (9) change infrastructure. Our multifaceted implementation strategies for the direct access model will consist of multiple discrete approaches. These strategies may operate at both the individual level (addressing knowledge, attitudes, and skills of adopters and implementers) and the organizational level (influencing institutional commitment and strong leadership). We will make reference to the CFIR guide to identify which ERIC implementation strategies would best address specific CFIR-based contextual barriers [29, 30].

### Implementation Research Logic Model (IRLM)

**Fig. 2:**
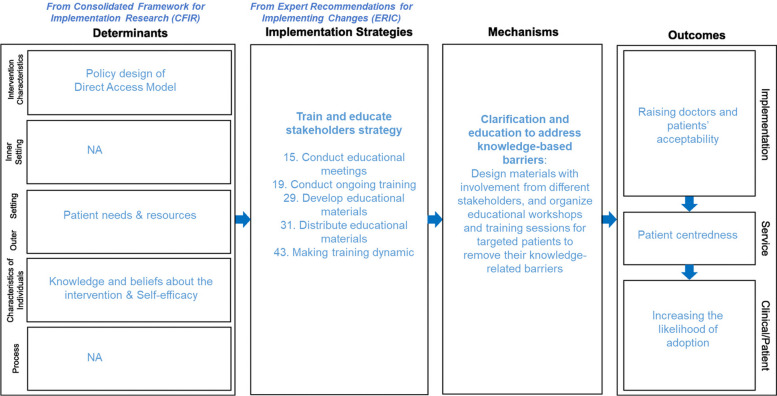
Implementation research logic model with example

Given the study’s complexity, which involves multiple tasks and research methods, we will use an IRLM to navigate this intricate landscape and guide the planning and execution of the study, and synthesis of findings [31] (Fig. 2). IRLM graphically depicts the complex methodological process, highlighting the relationships between implementation determinants, chosen strategies, mechanisms of action, and their impact on both implementation and clinical outcomes. The IRLM can facilitate the expert steering group and Delphi expert participants in developing the policy design and implementation strategies for physiotherapist direct access aligned with context-specific barriers and facilitators (implementation determinants). A critical first step is the development of likely acceptable intervention design of the policy from the options considered as this will define the characteristics intervention that can affect the implementation factors in the other domains and the subsequent implementation strategies. If there is no consensus for a single policy design, consensus will be sought for two or more options. The implementation strategies may have to be amended to tailor for each of them. The implementation strategies will work through specific mechanisms of action to drive changes within the context or influence the behaviors of those involved in the implementation. Implementation outcomes represent the proximal impacts of our chosen strategies and their associated mechanisms, which then relate to the overall effectiveness of the intervention.

For instance, when considering the determinant of “patients’ knowledge” using a deductive approach, we will match this factor to one of the CFIR domains, “Characteristics of individuals – Knowledge and beliefs about the intervention”. Drawing from the ERIC taxonomy, we will identify the “train and educate stakeholders strategy” as a means to address the knowledge gap. Other strategies involve conducting educational meetings and collaborative learning initiatives. The mechanisms of action associated with these strategies include designing targeted educational meetings for patients to remove their knowledge-related barriers. Ultimately, this approach can enhance acceptability and increase the likelihood of adoption – critical implementation outcomes.

These proposed strategies will be verified by Delphi expert participants to assess their validity, feasibility, and clarity. The IM framework, along with the CFIR and ERIC, as presented in the IRLM, is well-suited for this study. It facilitates a logical linkage of the successive methodological steps in understanding the policy intervention in context, defining the components in the design that are best suited to the local context, and analyzing determinants in the subsequent step that need to be considered for the implementation and strategy development.

### Delphi survey

The Delphi technique is a method for structuring a group communication process effectively, allowing a collective of individuals to address complex problems [32]. Various approaches exist for conducting the Delphi Survey, and the method is adaptable to specific study aims [33, 34]. The Group Delphi Technique structures a process of deliberation of the expert participants, enabling the sharing and understanding of the rich perspectives and diverse knowledge base of expert participants from different fields. This approach is well-suited for examining a broad and complex field and will be adopted in this study. Encouraging participants to express their viewpoints, disagreements, and reasoning is crucial to maximizing the benefits of the technique, especially given the challenge of maintaining participant anonymity.

The Delphi method has developed into a widely utilized approach within policy research, especially prominent in the domain of healthcare field and particularly valuable as a last stage of a multi-methods policy research study. The group Delphi can contribute to construct validity and generate new data and context specific knowledge or evidence [35]. The purpose of the Delphi Survey in this study is for deliberation and consensus-building on policy design and implementation of direct access to physiotherapists in Hong Kong. It addresses aspects of the policy design such as potential target patients for direct access, and preliminary scope and conditions for direct access (including sector and setting). The implementation strategies developed using the IRLM will also be assessed on their validity, feasibility, and clarity.

The sample size for the Delphi technique varies widely in the literature, typically falling within the low to medium double-digit range [32]. Selection criteria for Delphi expert participants include experience, professional credentials, and availability. A panel of 10 expert participants, including patients, physiotherapists, medical doctors, other related healthcare professionals, provider organizations, and academia with at least 5 years of experience across different work settings, will be formed. Incentives will be provided to compensate for their time. This number is consistent with the recommended optimum number of experts proposed by Baker et al. [36].

**Fig. 3:**
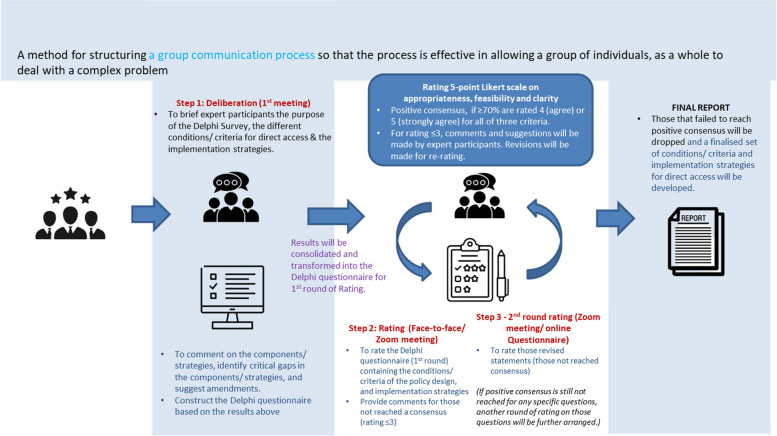
The Delphi survey process

The principal investigator will lead the Delphi process. Potential expert participants will receive email invitations with information about the research aim and details of the Delphi study. Upon expressing interest in participating, experts will receive the Delphi package, including a written consent form, rating criteria, and ground rules for the Delphi survey process. The Delphi Survey will comprise at least 3 rounds: (i) the first round (with a pre-deliberation session), (ii) the second round (face-to-face/ virtual meeting), and (iii) the third round (online self-administered questionnaire) (Fig. 3).

(i) The first round of the Delphi Survey (with pre-deliberation session)

We expect it might take several rounds to reach a consensus on the intervention design of the policy covering conditions/ criteria for direct access among the participants, due to the complexity of the subject, viz. diverse healthcare settings, varying professional perspectives, and the range of experience and competencies among physiotherapists, and public knowledge. The Delphi Survey plays a critical role in understanding potential controversies before the adoption of direct access. To facilitate an open exchange and encourage the expression of issues and viewpoints which may be considered controversial, the first round of the Delphi will be conducted in a virtual setting with anonymity between the group members. To facilitate this process, the first round of Delphi will begin with a deliberation session where participants will receive an overview of the Delphi Survey’s purpose and a set of conditions/ criteria for direct access developed based on the system of the existing literature and qualitative study findings and the expert working groups input. We will also introduce the barriers and facilitators associated with each option of the policy design and corresponding implementation strategies using the IRLM to outline the steps in the development process to facilitate their understanding and deliberation. After this session, expert participants will complete an online questionnaire (via Qualtrics) to identify critical gaps and amend both the components of the direct access model of the policy intervention and implementation strategies as needed. Based on the preliminary result from the anonymous real-time online questionnaire, the study team will further clarify and explain the conditions, criteria, and implementation strategies. These insights will then be consolidated and transformed into the Delphi questionnaire for the second round of the Delphi Survey.

(ii) The second round (face-to-face/ virtual meeting)

The second round of the Delphi process will involve a second virtual meeting where participants will rate the conditions and criteria for policy design and implementation strategies using a 5-point Likert scale on the acceptability, feasibility, and clarity. To facilitate the rating process, the principal investigator will first provide an initial overview of the questions before each participant completes the questionnaire individually and anonymously. Consensus will be considered positive if ≥ 70% of participants rate items with a score of 4 (agree) or 5 (strongly agree) across all three criteria. This threshold aligns with the robust standard and has been widely adopted [37–39]. Expert participants are encouraged to provide comments or suggestions for questions rated ≤ 3.

During the meeting, we will present the Delphi results to the expert participants, including anonymous comments related to the items not reaching consensus. The participants’ inputs will be sought on revising items not achieving positive consensus. These modified items will then be re-rated in the third Delphi round.

(iii) The third round (online self-administered questionnaire)

In the third round, expert participants will receive email invitations to rate the conditions/ criteria and implementation strategies that have not yet reached positive consensus via a self-administered online platform (Qualtrics). Expert participants will be asked to rate any new or modified conditions/ criteria and implementation strategies proposed during the second round. Participants are encouraged to provide remarks or suggestions for items rated ≤ 3.

Data analysis in the third round will follow a similar approach to that in the second round. If positive consensus remains elusive for specific questions, we will arrange one final round of rating for those conditions/ criteria and implementation strategies. Generally, three or more rounds of rating are preferable to achieve consensus [40]. At the conclusion of this process, any conditions/ criteria and implementation strategies that still fail to reach a positive consensus will be dropped, resulting in a finalized set of conditions/ criteria and implementation strategies for direct access.

## Data analysis

For the key informant interviews and focus group discussions, thematic analysis will be employed to identify, analyze, and report themes for each method. Dedoose software will be used for coding and managing the analysis. To enhance the reliability, a group analytical approach will be applied. Two research staff will independently create codes for 20% of the transcripts from each method. They will then cluster these codes into potential themes and sub-themes. Next, they will meet and agree on the potential codes and themes. As they continue reading the transcripts separately, they will repeatedly compare and contrast their coding schemas to identify new themes and codes. This iterative process will lead to a consensus-based interpretation of the data, ensuring consistency and a comprehensive understanding of the ideas presented during interviews and focus group discussions.

Recurrent themes that emerge among interviewees across and within different focus groups will be noted, merged, and refined under a master theme as the analysis progresses. Interpretations of these themes will be illustrated with extracts from the transcripts. The analysis will follow these steps: (1) study the transcripts, (2) generate initial codes, (3) search for themes, (4) review themes, (5) define and name themes, and (6) produce the report [24]. We will adhere to the Consolidated Criteria for Reporting Qualitative Research (COREQ), a 32-item checklist for interviews and focus groups [41].

Regarding the Delphi Survey, consensus on the conditions/ criteria and implementation strategies will be reached if ≥ 70% of expert participants rate them as 4 (agree) or 5 (strongly agree) on a 5-point Likert scale. We will calculate the median rating for each item across the three evaluation criteria instead of mean values to mitigate the influence of outliers. Standard deviation and interquartile range values will be calculated to reflect the magnitude of agreement or disagreement amongst participants.

## Discussion

This study aims to examine the policy design for direct access to physiotherapists in Hong Kong, including conditions, settings, training considerations, and competence requirements necessary to facilitate direct access. Additionally, we seek to identify the associated barriers and facilitators impacting policy implementation. Ultimately, our goal is to inform the development of an acceptable policy design and a robust implementation strategy to achieve the policy objective.

Given the specialized nature of this topic, the establishment of an Expert Steering Group comprising experts is crucial for shaping the policy design and the implementation strategies for physiotherapy direct access. This collaborative process aims to ensure a well-informed and effective implementation strategy.

Additionally, leveraging expert contributions with Delphi methodology during the final stages of research can offer several valuable functions. Firstly, elicitation of tacit knowledge: The qualitative insights provided by the Delphi expert group are particularly valuable due to the diverse expertise of its participants. Serving as an additional conduit for gathering new evidence or arguments, the Delphi Group can significantly enrich and deepen the analysis by constructively challenging initial findings, requiring the analysis to be inductively revised and expanded to incorporate these divergent perspectives [42]. Secondly, facilitation of communication with key stakeholders: Engaging a policy-oriented Delphi Group can be especially instrumental in bridging the research-to-policy gap highlighted in extensive literature [43]. The process of developing a set of evidenced informed policy design options for submission to the Delphi Group inherently enhances accessibility of research findings, facilitating their utility for policy-makers [42]. Thirdly, deliberation on policy recommendations: The design of the policy itself, informed by these expert insights, can significantly influence the implementation process and its outcomes. The Delphi method is particularly advantageous as some panelists may belong to the policy community and possess greater expertise in framing policy recommendations than researchers [42]. The findings also complement the intervention characteristics domain of the CFIR by incorporating a policy perspective from the expert panelists.

In Hong Kong, physiotherapists and medical doctors hold divergent views on the direct access model, a topic that has garnered attention in the literature [11, 15, 16, 44]. In alignment with the Chief Executive's 2021 Policy Address, which proposed a legislative amendment to allow a direct access model [18], the Physiotherapists Board of Hong Kong established an interdisciplinary Working Group on Direct Access [45] comprising doctors, physiotherapists, and community representatives. The Group proposed a model that permits patients to access physiotherapy without a doctor's referral in private primary care settings, featuring different practice limits and periods for patients with or without pre-existing medical diagnoses [45]. In December 2023, the Government put forth another direct access model based on the collected views, outlining three specific circumstances where direct access is permitted, proof of diagnosis, compliance with clinical protocols, and clear definitions of emergencies and other circumstances. Given the contrasting opinions among doctors and physiotherapists, achieving greater consensus among stakeholders is challenging. A Delphi survey involving a panel of experts representing various stakeholders can help mitigate differences and lead to a more consensus-driven design and implementation of direct access [17]. Due to the complexity of the subject arising from varying professional perspectives and knowledge gaps of different stakeholders, a better engagement in a deliberative process will enable clarification of each other’s positions and facilitate mutual understanding of each other’s perspectives, role, competency and ability, which could enhance the acceptability and adoptability of the direct access model.

## Conclusion

This study employs a sequential mixed-method approach to examine the policy design and implementation of direct access to physiotherapists in Hong Kong. The literature review will be continuously updated to assimilate emerging insights, aiding the development of policy frameworks and implementation strategies. Through key informant interviews and focus groups, we will identify barriers and facilitators related to the implementation of the direct access model, guided by the CFIR. Leveraging the ERIC taxonomy, initial implementation strategies will be identified, serving as a foundation for further exploration and refinement. Additionally, the IRLM will synthesize findings, facilitating consensus-building for informed policy design and implementation strategies. Ultimately, our study aims to facilitate access to physiotherapy services in Hong Kong by integrating research insights into acceptable policy design and actionable strategies for policy implementation, with expert and stakeholder inputs for a Delphi Survey playing crucial roles in shaping the recommendations.

## Supplementary Information

Supplementary Material 1.

## Data Availability

Not applicable.

## Abbreviations

CFIR: Consolidated Framework for Implementation Research
ERIC: Expert Recommendations for Implementing Change
IM: Implementation Mapping
IRLM: Implementation Research Logic Model
