# Supplementary material for: A study protocol for the policy intervention design and development of the implementation strategies for direct access to physiotherapists in primary care: a sequential mixed-method study using implementation mapping and a Delphi survey
Source: Implement Sci Commun. 2024 Dec 18;5:141. doi: 10.1186/s43058-024-00680-y (PMC11657651; doi:10.1186/s43058-024-00680-y)
Supplement: Supplementary file 1 — Supplementary Material 1. [file 43058_2024_680_MOESM1_ESM.docx]

Additional File 1:

**Study Conceptual Framework**

The evidence for the safety and effectiveness of direct access to physiotherapist has been studied in different health systems’ settings of providers and under different policy designs which prescribe conditions for the practice model that set conditions and limits on the disease nature, time or visits. There are significant evidence gaps to inform the adoption in the context in the health system of a country or jurisdiction. It is recognized that for many health systems and policy interventions, evidence is rarely complete and is uncertain, complex and contestable. A deliberative process is likely to be required to appraise and interpret the evidence and the application in the context of the intended policy. The deliberative process of expert stakeholders can combine research evidence with contextual evidence and expert (colloquial) evidence to inform policy decisions in the design [1]. An evidenced informed policy intervention will contribute to the acceptability in the implementation.

IM is a systematic process that guides the use of theory, empirical evidence, and stakeholder input to develop implementation strategies. Originating from Intervention Mapping, IM specifically expands upon Step 5 which involves “*planning for adoption, implementation, and sustainability of the program in real-life contexts by identifying program users and supporters and determining what their needs are and how these should be fulfilled”* [2]. IM enhances this approach by incorporating insights from Implementation Science, such as using CFIR to identify critical behaviors and contextual factors, ensuring the effectiveness of the selected implementation strategy by linking them to the identified influential factors. This systematic process enhances the reproducibility of our research, and the application of theories which elucidate the mechanisms behind the implementation strategies [3]. IM encompasses five key steps (Figure 1). First, an implementation needs assessment is conducted to identify program adopters and implementers by roles, and potential barriers and facilitators to implementation (Step 1). Second, the implementation outcomes, performance objectives, and determinants that create change matrices are identified and mapped (Step 2). These determinants will be mapped to CFIR’s five domains: intervention characteristics, outer setting, inner setting, characteristics of individuals, and the process [4]. We chose the CFIR because it is a meta-framework encompassing intervention characteristics and a wide range of influencing factors, enabling us to identify implementation determinants of the direct access model across multiple levels. The 5 domains of CFIR interact on complex ways which affect intervention effectiveness. In particular, the design of the intervention will determine the intervention characteristics, intervention source, evidence strength and quality, relative advantage, adaptability, trialability, complexity, design quality and packaging and cost. These intervention characteristics in turn could generate barriers and facilitators in the other 4 domains. Next, the implementation strategies will be selected or designed (Step 3). Subsequently, there should be a production of implementation protocols and materials (Step 4), followed by the evaluation of outcomes (Step 5). Given that government decisions on the final policy design and implementation, including a specific timetable for the direct access model are still pending, we anticipate that observable outcomes may not be available before the study concludes. Therefore, our study will primarily focus on Steps 1-3, while listing Steps 4 and 5 here ensures comprehensiveness and provides a reference for future decision-making by the government regarding implementation activities, resources, and evaluation.

**References**

1. Canadian Health Services Research Foundation. Conceptualizing and Combining Evidence for Health System Guidance. 2005.

2. Eldredge LKB, Markham CM, Ruiter RAC, Fernández ME, Kok G, Parcel GS: Planning Health Promotion Programs: An Intervention Mapping Approach: Wiley; 2016.

3. Waltz TJ, Powell BJ, Fernandez ME, Abadie B, Damschroder LJ: Choosing implementation strategies to address contextual barriers: diversity in recommendations and future directions. Implement Sci 2019;14(1):42.

4. Damschroder LJ, Aron DC, Keith RE, Kirsh SR, Alexander JA, Lowery JC: Fostering implementation of health services research findings into practice: a consolidated framework for advancing implementation science. Implement Sci 2009;4:50.
